# Dynamics of Fecal microRNAs Following Fecal Microbiota Transplantation in Alcohol-Related Cirrhosis

**DOI:** 10.3390/jcm14248623

**Published:** 2025-12-05

**Authors:** Cristian Ichim, Adrian Boicean, Samuel Bogdan Todor, Ioana Boeras, Paula Anderco, Victoria Birlutiu

**Affiliations:** 1 Faculty of Medicine, “Lucian Blaga” University of Sibiu, 550024 Sibiu, Romania; cristian.ichim@ulbsibiu.ro (C.I.); paula.anderco@ulbsibiu.ro (P.A.); victoria.birlutiu@ulbsibiu.ro (V.B.); 2 Biology and Ecology Research Center, Faculty of Sciences, “Lucian Blaga” University of Sibiu, 550012 Sibiu, Romania; ioana.boeras@ulbsibiu.ro

**Keywords:** micro-RNA, fecal microbiota transfer, alcohol-related liver cirrhosis, biomarkers

## Abstract

**Background:** Micro-RNAs (miRNAs) are emerging as pivotal regulators of pathophysiological processes, reflecting systemic responses to stress, inflammation and metabolic imbalance. Their role in advanced liver disease and in modulating responses to therapeutic interventions, such as fecal microbiota transfer (FMT), remains insufficiently characterized. **Methods:** We conducted a prospective study including six male patients with toxic ethanolic liver cirrhosis undergoing FMT and six healthy controls. Stool and blood samples were collected pre- and post-FMT. Fecal micro-RNA expression (miR-21, miR-122, miR-125, miR-146 and miR-155) was quantified using RT-qPCR and normalized to miR-26c. Associations with noninvasive fibrosis markers (FIB-4, APRI, elastography, CAP) and biological parameters were analyzed through multivariable regression and Pearson correlation, with internal validation by bootstrapping. **Results**: One week after fecal microbiota transfer, miR-21 and miR-146 exhibited significant expression changes, while miR-122, miR-125, and miR-155 showed non-significant trends toward increased expression. Post-FMT increases in miR-21, miR-122, miR-146 and miR-155 were consistently associated with reductions in hepatic fibrosis markers (FIB-4, APRI and liver stiffness), whereas no significant associations were observed with CAP. **Conclusions**: Fecal micro-RNAs reflect interconnected molecular networks that capture systemic adaptations to FMT. Despite a limited cohort, these findings highlight their potential as integrative biomarkers and as therapeutic targets in advanced liver disease. Larger-scale studies are warranted to validate clinical utility.

## 1. Introduction

Liver cirrhosis is the irreversible outcome of chronic liver injury, characterized by progressive fibrosis, distortion of hepatic architecture and impaired liver function [1,2]. It represents a significant global health burden, accounting for substantial morbidity and mortality, with alcohol-related cirrhosis being one of the most frequent etiologies [3,4]. In Central and Eastern Europe, where harmful alcohol consumption remains highly prevalent, toxic ethanolic cirrhosis continues to be a leading cause of end-stage liver disease, imposing a considerable socioeconomic and healthcare impact [5]. Despite advances in supportive management, therapeutic options remain limited and liver transplantation is often the only definitive intervention [6]. This highlights the urgent need for novel diagnostic tools and alternative treatment strategies capable of modulating disease progression.

In recent years, the gut–liver axis has attracted growing attention as a key player in the pathophysiology of cirrhosis [7]. Intestinal dysbiosis has been shown to exacerbate hepatic injury, systemic inflammation and metabolic imbalance, creating a self-perpetuating cycle of organ dysfunction [8,9]. Fecal microbiota transfer (FMT) has emerged as a promising therapeutic strategy aimed at restoring microbial homeostasis [10]. While most extensively studied in recurrent *Clostridioides difficile* infection, FMT is increasingly investigated in chronic liver disease, where it may exert beneficial effects by modulating intestinal permeability, immune activation and systemic metabolic signaling [11,12]. However, the molecular mediators linking FMT to clinical outcomes in cirrhotic patients remain poorly understood.

MicroRNAs (miRNAs) are small, non-coding RNA molecules with critical regulatory functions in gene expression at the post-transcriptional level [13,14]. They are now recognized as central players in diverse physiological and pathological processes, including hepatic fibrosis, metabolic regulation, immune modulation and tumorigenesis [15,16]. Their remarkable stability in biological samples, including stool, makes them attractive candidates as noninvasive biomarkers [17]. Among them, miR-21, miR-122, miR-125, miR-146 and miR-155 have been consistently implicated in hepatic injury, cardiovascular and renal dysfunction, inflammation and lipid metabolism [18,19,20,21]. These molecules not only reflect the systemic impact of cirrhosis but may also provide insights into therapeutic response following interventions such as FMT.

Building on these premises, the present study investigated fecal miRNA expression in patients with toxic ethanolic liver cirrhosis undergoing FMT, compared with healthy controls. By integrating molecular analysis with clinical and biochemical parameters, including noninvasive fibrosis markers, we sought to elucidate the potential role of miRNAs as integrative biomarkers of disease activity and response to microbial modulation. Understanding these associations could pave the way for novel diagnostic strategies and targeted therapeutic approaches in advanced liver disease.

## 2. Materials and Methods

### 2.1. Study Groups and Eligibility Criteria

The study enrolled adult patients admitted to the Sibiu County Clinical Emergency Hospital, mainly from the Department of Gastroenterology, who had been diagnosed with toxic ethanolic liver cirrhosis. We included patients regardless of Child–Pugh stage. However, the application of our key inclusion criterion—verified post-FMT alcohol abstinence through a relative or close contact—led to the exclusion of several individuals. Nevertheless, the baseline characteristics did not show statistically significant differences between the Child–Pugh severity classes. Patients under 18 years of age, those diagnosed with cirrhosis of an etiology other than alcohol consumption, patients with uncertain or unconfirmed diagnosis, as well as those with associated oncological conditions or major trauma that could have influenced the study results, were excluded. Also excluded were patients who refused to participate in the study and sign the informed consent, as well as those in a coma or with severely altered consciousness. Although this characteristic was not intentionally imposed, all individuals included in the study were male, a fact that adds value by further narrowing the cohort and excluding potential variations resulting from sex-related differences. Once they consented to participate in the study, individuals were integrated into a specific protocol adapted to the category in which they were included.

Patients with cirrhosis selected for fecal microbiota transfer (n = 6) were first clinically and paraclinically evaluated. A physical examination, collection of biological samples (stool and blood), as well as imaging and elastographic evaluations, were performed. After the initial assessment, a complete colonoscopy up to the cecum was carried out, with fecal microbiota transfer performed at this level. The transfer product was obtained from a healthy donor. Starting from the moment of fecal microbiota transfer, patients were dynamically evaluated at approximately 7-day intervals. Variations in the interval between FMT and reevaluation were permitted, depending on the patient’s availability and the possibilities of the medical service. All evaluations included clinical examination, ultrasound and blood sampling for laboratory analyses. Specifically, at the reevaluation following FMT, a stool sample was collected for micro-RNA analysis. Patients in this category were included in the study only in the absence of antibiotic use in the last three months. During the study, alcohol consumption and the administration of any type of antibiotics (including rifaximin) were prohibited.

Patients in the control group of healthy individuals (n = 6) consisted of volunteers without digestive pathologies or other relevant chronic conditions and who had not received antibiotic treatment in the last three months. These participants gave their consent to take part in the study and provided stool samples required for comparative micro-RNA analysis, carried out against patients who underwent fecal microbiota transfer.

### 2.2. Fecal Donor Selection and Preparation Protocol

Fecal donors were selected both from relatives of the patients who underwent transfer and from unrelated individuals. Young donors without comorbidities who expressed written consent for voluntary participation were chosen for this procedure. They underwent rigorous clinical and paraclinical evaluation, aimed at eliminating any risk of contamination and the selection process also included the completion of a mandatory questionnaire designed to exclude donors with potential epidemiological risk capable of transmitting infectious diseases or other pathologies to patients with liver cirrhosis.

On the morning of each procedure, donors prepared the material intended for transfer according to a standardized protocol, which had been provided to them beforehand, at the time of donation acceptance. The preparation protocol used was adapted from the model implemented in the Gastroenterology Clinic of Sibiu for patients diagnosed with *Clostridioides difficile* and was rigorously followed by all donors. The preparation model is described below:

Fecal material must be collected in the morning of the transplant and must not be stored for more than 6 h (otherwise the sample becomes ineffective).

The required quantity is at least 70 g of stool, which is then mixed in a jar with 250 mL of physiological saline solution (NaCl 0.9%). The mixing must last at least 3 min.

In the next stage, the mixture in the jar is filtered through a gauze sieve (2–3 layers) and the filtrate is the material brought to the hospital for transplantation.

Colonoscopy was performed according to the standard protocol, using polyethylene glycol-based solutions for intestinal preparation. All patients were analgo-sedated during the entire procedure. To optimize the effects of fecal microbiota transfer, patients were instructed to avoid eliminating the transplanted content for at least two hours post-intervention. During the study, no other medications likely to influence the research results were administered.

### 2.3. Sample Handling and Statistical Methods

For the purpose of micro-RNA analysis, six patients diagnosed with toxic ethanolic liver cirrhosis, who had been prepared for fecal microbiota transfer, were selected. Prior to undergoing fecal microbiota transfer, a stool sample was collected from each patient on the same day as the blood analyses. Additionally, at one week post-transfer, during the first reevaluation, another stool sample was collected. These two samples were processed as soon as possible after collection and stored until the time of laboratory analysis.

Sample preparation was carried out primarily by introducing the stool into 1.5 mL Eppendorf microtubes with caps, under conditions as sterile as possible. The sterility of handling biological samples refers to avoiding any external contamination that might alter the results. After placing the material into the tubes, they were weighed using high-precision scales. An effort was made to ensure that all samples were approximately identical in terms of the quantity of biological material, maintained at around 60–70 g. In order to ensure prolonged storage and optimal preservation of RNA 1 mL of TRIzol Reagent was added to the stool sample, as per the manufacturer’s instructions.

After homogenization of fecal matter with TRIzol, the samples were weighed again, sealed, numbered, homogenized using a homogenizer and promptly stored in a freezer at −80 °C, this being the optimal temperature for preserving micro-RNA. It is important to note that the tubes, pipettes and all instruments that came into contact with the patient’s stool were carefully selected to avoid any possibility that enzymes or foreign substances could degrade the micro-RNA. Storage was maintained until all samples from all patients were collected, so that laboratory analysis could be performed simultaneously, thereby minimizing potential processing errors. Stool samples from healthy individuals were collected only once, then prepared and stored identically to those of the experimental group.

Once all samples were collected, they were thawed and processed simultaneously. In total, 18 samples were analyzed: 6 collected pre–fecal microbiota transfer, 6 collected post–fecal microbiota transfer and 6 from the control group of healthy individuals.

For the purpose of validating the eligibility of fecal micro-RNA measurements, miR-26c was selected as the reference gene, demonstrating stable expression without significant differences between the control group and the study group (Mann–Whitney U test, *p* = 0.589).

Following the normalization process, gene expression was quantified relative to the endogenous reference gene (miR-26c), which was used as the normalizer. The ΔCT value was calculated according to the formula:

ΔCT = CT target gene − CT miR-26c

where CT target gene represents the arithmetic mean of the three measurements for the gene of interest and CT miR-26c is the mean of the CT values corresponding to the reference gene within the same sample [22].

This value was determined for each evaluation time point (pre-transplant and one week post-transplant). To quantify the variation in expression between these two moments, the ΔΔCt difference was calculated:

ΔΔCt = ΔCt post-transplant (1 week) − ΔCt pre-transplant

The fold change in miRNA expression in the study group compared to the control group was calculated using the delta delta CT method [23].

Fold change = 2^−ΔΔCT^

In the final stage of the analysis, the correlation between the dynamic variation in the expression of the studied micro-RNAs and the changes in noninvasive hepatic fibrosis markers (FIB-4, APRI, liver elastography [E, kPa] and CAP [dB/m]) was investigated. The variation in these markers was calculated according to the formula:

Δ Marker = M1 − M0

where M0 represents the marker value before transplantation and M1 its value one week after transplantation.

### 2.4. RNA Extraction and miRNA Quantification by RT-qPCR

Total RNA was extracted from the samples using the TRIzol Reagent (Invitrogen/ThermoFisher Scientific, Waltham, MA, USA) according to the manufacturer’s recommendations and RNA was resuspended in a final volume of 100 µL ultrapure water. After extraction, miRNA quantity and quality were verified by fluorometry with Qubit miRNA Assay kit and a Qubit 4 Fluoreometer (ThermoFisher Scientific, Waltham, MA, USA). For quantification of the select miRNA molecules by RT-qPCR, the TaqMan Advanced miRNA Assay kit (ThermoFisher Scientific, Waltham, MA, USA) was used. The assay comprises two parts, the first is the synthesis of cDNA and the second is quantification of miRNA by real-time PCR. cDNA synthesis is also divided into multiple steps: addition of a polyA tail, followed by adaptor ligation, the reverse transcription reaction and amplification of the RT products by PCR. All steps were performed according to the protocol starting with a volume of 2 µL of total RNA for each sample. The miRNAs of interest were then quantified by real-time PCR using specific primers and probe labeled with FAM-TAMRA, the TaqMan Fast Advanced Master Mix, and 5 µL of cDNA template in a QuantStudio 5 thermocycler (AppliedBiosystems by ThermoFisher Scientific, Waltham, MA, USA). Cycling conditions were 95 °C for 20 s followed by 40 cycles of 95 °C for 1 s and 60 °C for 20 s. Amplifications were run in triplicate for all samples.

The sequence of laboratory analysis comprised numerous steps that were carefully followed and the entire process of sample analysis was conducted in accordance with the protocols recommended by the manufacturers. The advanced analysis method (TaqMan™ Advanced) was selected for a simpler and clearer comparative evaluation relative to the classical method, in which the risk of errors is slightly higher. The entire process was designed for the quantitative assessment of micro-RNA results (obtained from stool samples) detected by RT-qPCR. The selected micro-RNA types were:

- Hsa-miR-21-5p;
- Hsa-miR-122-5p;
- Hsa-miR-125-5p;
- Hsa-miR-146-5p;
- Hsa-miR-155-5p.

### 2.5. Statistical Analysis

Fold changes in micro-RNA expression (Table 1) are presented as median and interquartile range (IQR). In Table 2, associations between liver fibrosis markers and micro-RNAs at baseline (pre-FMT) were assessed using multivariable logistic regression, with B-coefficients, *p*-values and 95% confidence intervals reported. Table 3 summarizes pre- and post–fecal microbiota transfer (FMT) micro-RNA fold change, expressed as median (IQR), and differences were evaluated using Friedman two-way ANOVA. For Table 4, a multivariable linear regression was performed, with results reported as B-coefficients along with 95% confidence intervals. Table 5 and Table 6 report Spearman correlation coefficients, with statistical significance indicated by an asterisk (*). For all analyses, α = 0.05 and statistical analyses were performed using IBM SPSS version 26.0.

## 3. Results

### 3.1. Fold Change Results

The fold change in miRNA expression in the study group and the control group was determined using the ΔΔCT method, yielding the following results:

One week after fecal microbiota transfer, circulating micro-RNA levels showed variable fold changes. MiR-21 and miR-125 had relatively low median fold changes (0.384 and 0.386, respectively), indicating minimal increases or slight decreases in expression. In contrast, miR-122, miR-146, and miR-155 exhibited higher median fold changes (1.704–1.739), reflecting a more pronounced upregulation.

### 3.2. Correlation Between micro-RNAs and Hepatic Fibrosis Markers Pre-Transplant

The following are the correlations assessed by multivariable linear regression between the fold change in expression of miRNA in patients of the investigated microRNAs (miR-21, miR-122, miR-125, miR-146, miR-155) and the values of noninvasive hepatic fibrosis markers: FIB-4, APRI, liver elastography expressed in kilopascals (E, kPa) and controlled attenuation parameter (CAP, dB/m) (Table 2).

Table 2 shows the associations between hepatic fibrosis markers and the fold change in the studied micro-RNAs at baseline. MiR-21 was significantly negatively correlated with both FIB-4 (B = −0.003, *p* = 0.010) and APRI (B = −0.001, *p* = 0.019), suggesting higher miR-21 levels are associated with lower fibrosis scores. For liver stiffness (E, kPa), none of the micro-RNAs showed significant correlations and confidence intervals were wide, indicating no clear relationship. In contrast, miR-122 (B = 51.690, *p* = 0.044) and miR-146 (B = 60.078, *p* = 0.035) were significantly positively associated with CAP. The remaining micro-RNAs showed no significant associations across the fibrosis or steatosis markers, although some trends were observed, highlighting potential but non-significant relationships.

### 3.3. Expression of microRNAs at One Week Post–Fecal Microbiota Transfer

**Figure 1:**
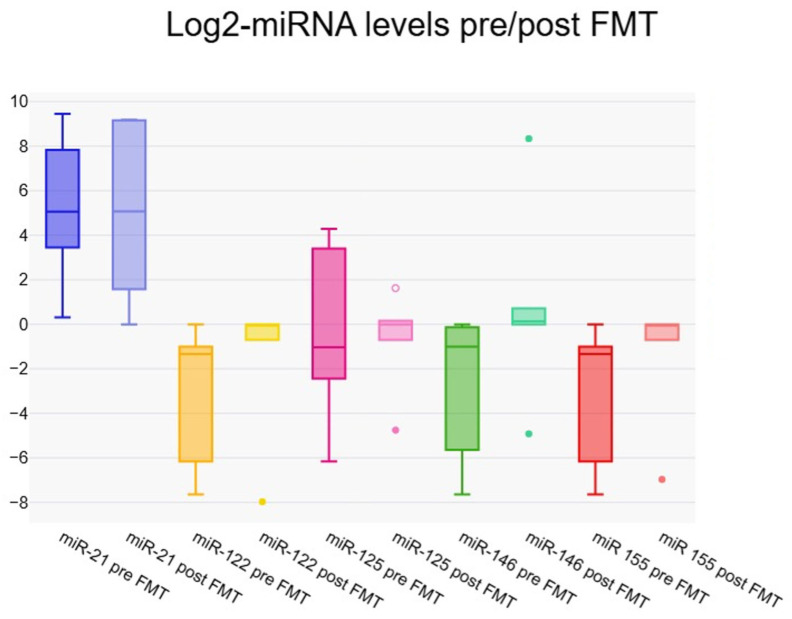
Boxplot representing pre- and post-FMT (1 week) values of Log2 fold change-miRNA levels.

One week after fecal microbiota transfer, several micro-RNAs showed notable changes compared to baseline. MiR-21 and miR-146 exhibited statistically significant increases (*p* = 0.021 and *p* = 0.002, respectively), indicating a clear upregulation post-transplant. MiR-122, miR-125 and miR-155 also showed increases in median levels, though these did not reach conventional significance (*p*-values 0.058–0.068), suggesting a trend toward upregulation. Overall, the data indicate that FMT can modulate circulating micro-RNA levels within one week, with the most pronounced and significant effects observed for miR-21 and miR-146 (Table 3) (Figure 1).

### 3.4. Dynamic Changes in micro-RNA Expression at One Week Post–Fecal Microbiota Transfer

A negative variation indicates a decrease in fibrosis markers, suggesting improvement or regression of hepatic fibrosis, whereas a positive variation indicates an increase in markers, suggesting fibrosis progression. Subsequently, the relative fold change was identified using the formulas described in the Section 2.

The multivariable linear regression analysis with internal validation by bootstrapping examined the associations between post-FMT micro-RNA expression and changes in hepatic fibrosis markers.

A consistent pattern of significant negative associations emerged for several micro-RNAs—particularly miR-21, miR-122, miR-146 and miR-155—across multiple fibrosis indices. For the FIB-4 and APRI scores, these micro-RNAs showed statistically significant inverse relationships (*p* < 0.05), indicating that higher post-FMT expression levels were linked to decreases in fibrosis severity. MiR-125, by contrast, did not show any significant correlation with these indices (*p* > 0.05).

Similar trends were observed for liver stiffness (E, kPa), where miR-21, miR-122, miR-146 and miR-155 again displayed significant negative coefficients, suggesting a consistent relationship between increased expression of these micro-RNAs and reductions in liver stiffness. Notably, miR-146 showed one of the strongest associations (B = −0.011, *p* = 0.004).

In contrast, none of the studied micro-RNAs demonstrated significant correlations with CAP (dB/m), implying that their effects are more closely related to fibrosis than to hepatic steatosis.

The Pearson correlation analysis shows that several micro-RNAs are significantly associated with specific biological markers. MiR-21 was strongly negatively correlated with CA 19-9 (r = −0.709) and CEA (r = −0.587), and positively with AFP (r = 0.647), suggesting inverse relationships with certain tumor markers but a direct relationship with AFP. MiR-122 showed significant positive correlations with CEA (r = 0.690) and monocyte count (r = 0.606), while miR-125 was also positively correlated with monocyte count (r = 0.598), indicating potential links with immune activity. MiR-146 was significantly positively associated with free T4 (r = 0.670), suggesting a relationship with thyroid function. Finally, miR-155 showed a strong positive correlation with ESR (r = 0.681), highlighting a possible link with systemic inflammation (Table 5).

At one week post-FMT, several significant associations were observed between micro-RNAs and biological markers. MiR-21 was negatively correlated with liver, kidney function markers and hematological markers (ALT, creatinine, iron, AFP, leukocytes, neutrophils; r = −0.600 to −0.943) and positively with folic acid (r = 0.771). MiR-122 showed positive correlations with amylase, lymphocytes, PDW and a strong negative correlation with GGT (r = −0.829). MiR-125 was positively associated with total proteins, lymphocytes, platelets, ESR and negatively with magnesium and MPV. MiR-146 correlated positively with cholesterol, LDL, macroplatelets and negatively with IgG, IgM, CRP, and ferritin. MiR-155 was positively associated with amylase, lymphocytes, and PDW, and negatively with GGT. These results indicate that post-FMT micro-RNA expression is linked to multiple metabolic, inflammatory and hematologic parameters, highlighting broad systemic effects (Table 6).

## 4. Discussion

At one week post-FMT, the analysis of micro-RNA expression and their correlations with a wide range of biological markers revealed a complex landscape of molecular regulation and systemic responses following the procedure. The data suggest that micro-RNAs act as integrative regulators of organ function, metabolism and immune-inflammatory pathways in cirrhotic patients, with each molecule reflecting distinct physiological domains.

miR-21 emerged through its associations with hepatobiliary markers (total bilirubin, ALT), renal function (creatinine), thyroid hormone parameters (FT4, TSH) and metabolic status (BMI). These results point toward a central role of miR-21 in the adaptive modulation of metabolism and systemic homeostasis, integrating organ dysfunction with systemic responses to metabolic stress. Our findings indicate potential physiological relevance, but not sufficient justification for promoting microRNAs—including miR-21—as clinically actionable biomarkers without further large-scale and longitudinal studies. [21,24,25]. Other studies emphasized its role within the heart–kidney axis and the therapeutic strategies targeting this pathway in cardiac and renal failure [25,26,27].

miR-122 showed significant correlations with pancreatic markers (amylase) and lymphocyte count. These associations underline its involvement in both pancreatic activity and systemic inflammation. The findings resonate with previous studies highlighting miR-122 as a key player in fibrogenesis and cellular stress responses, supporting its relevance as a biomarker of inflammation and tissue injury. [28,29,30,31]. In our cohort, its association with acute-phase proteins suggests that miR-122 may capture subclinical inflammatory activity and systemic immune modulation following FMT.

miR-125 correlated significantly with inflammatory markers (CRP, ESR) and hematological parameters (platelet count, PDW). These results are consistent with the literature pointing to the role of miR-125 in immune regulation and platelet biology [32,33,34]. The involvement of the miR-125 family in cardiovascular development and immune responses was also described [35]. The correlations observed in this study suggest that miR-125 may participate in systemic inflammatory and hematological responses in cirrhotic patients after FMT. However, these associations are preliminary and should not be interpreted as evidence of a direct regulatory role.

miR-146 demonstrated a distinct pattern, being positively associated with lipid metabolism (total cholesterol, HDL-cholesterol, LDL-cholesterol) while showing inverse correlations with several inflammatory and immunological markers (CRP, ESR, IgG, IgM, ferritin, vitamin B12, NLR). This dual profile suggests a role as a modulatory regulator at the interface between lipid homeostasis and immune control [19,36,37,38]. Its importance in HDL metabolism and reverse cholesterol transport was also reported [39]. Within the present context, miR-146 may contribute to maintaining energy balance while dampening excessive immune activation, which is particularly relevant in cirrhotic patients at high risk of systemic inflammation.

miR-155 correlated with lymphocyte count, PDW and amylase, suggesting a brief immune–inflammatory response in the early post-FMT phase. Although previous studies have shown that miR-155 increases in systemic inflammatory states, these data remain exploratory and do not substantiate a defined mechanistic function [40].

Altogether, these findings suggest that the analyzed microRNAs may reflect different facets of post-FMT physiology: miR-21 appears linked to metabolic and organ-related changes, miR-122 and miR-125 to inflammatory status, miR-146 to lipid–inflammatory balance and miR-155 to early immune activation. The observed correlations indicate coordinated molecular responses rather than isolated biomarker functions, pointing to interconnected regulatory networks that mirror the patient’s overall clinical state.

It is important to emphasize that not all correlations reached statistical significance. For instance, despite upward post-FMT trends in several micro-RNAs (including miR-122 and miR-155), most failed to achieve significance, likely due to the limited sample size and short follow-up period. Nonetheless, the relatively low *p*-values observed suggest biologically relevant tendencies that may become significant in larger cohorts.

## 5. Limitations and Future Perspectives

From a translational perspective, the study suggests that microRNAs may have potential as integrative biomarkers capable of reflecting systemic responses beyond conventional biochemical markers. Their monitoring could provide preliminary insights into patients’ responses to FMT, although further validation in larger cohorts and with longer follow-up is required. Similarly, the observed correlations with fibrosis-related indicators (including scoring systems and elastography measurements) are exploratory and should be interpreted cautiously. While these findings raise the possibility that microRNAs might serve as future candidates for noninvasive monitoring tools, more extensive research is necessary to confirm their diagnostic or prognostic utility.

One limitation of this pilot study is the short one-week follow-up interval, which does not allow evaluation of stable clinical or fibrosis-related changes; however, a planned 3 and 6-month post-FMT follow-up will address this in future analyses.

## 6. Conclusions

This study highlights relevant associations between microRNA expression and key biochemical and clinical parameters in patients with toxic ethanolic liver cirrhosis undergoing fecal microbiota transfer. The identified microRNA patterns—miR-146 in relation to early metabolic–inflammatory shifts, together with miR-21, miR-122, miR-125 and miR-155 reflecting hepatic, inflammatory, metabolic and immune dynamics—suggest that these molecules may mirror coordinated physiological adjustments following the intervention. Rather than establishing any definitive biomarker function, these findings suggest that the observed microRNA patterns reflect participation in broader, interconnected regulatory networks that adapt dynamically to post-FMT physiological shifts.

## Figures and Tables

**Table 1:** Fold change variation after 1 week of FMT- median (IQR).

Variable	Fold Change
**miR-21**	0.384 (0.375–0.832)
**miR-122**	1.739 (1.000–2.009)
**miR-125**	0.386 (0.164–0.498)
**miR-146**	1.704 (1.378–2.009)
**miR-155**	1.739 (1.000–2.009)

**Table 2:** Correlations between hepatic fibrosis markers and the fold change in the studied micro-RNAs at baseline (pre–fecal microbiota transfer).

Variable	B-Coefficient	*p*-Value	95% CI for B
**FIB-4**			
**miR-21**	−0.003	0.010 *	(−0.006–−0.002)
**miR-122**	0.559	0.557	(−2.086–2.159)
**miR-125**	0.063	0.120	(0.007–0.124)
**miR-146**	0.427	0.649	(−1.204–2.221)
**miR-155**	0.559	0.541	(−1.862–2.178)
**APRI**			
**miR-21**	−0.001	0.019 *	(−0.002–−0.001)
**miR-122**	0.172	0.470	(−0.584–0.576)
**miR-125**	0.018	0.128	(0.005–0.028)
**miR-146**	0.080	0.721	(−0.318–0.518)
**miR-155**	0.172	0.491	(−0.558–0.564)
**E (kPa)**			
**miR-21**	0.003	0.145	(−0.006–0.002)
**miR-122**	−2.318	0.212	(−10.652–0.347)
**miR-125**	0.065	0.302	(−0.040–0.205)
**miR-146**	−1.642	0.145	(−3.670–2.187)
**miR-155**	−2.315	0.210	(−10.248–0.459)
**Cap (dB/m)**			
**miR-21**	−0.009	0.778	(−0.188–0.045)
**miR-122**	51.690	0.044 *	(3.544–110.521)
**miR-125**	3.421	0.086	(1.968–8.614)
**miR-146**	60.078	0.035 *	(17.412–104.878)
**miR-155**	51.690	0.052	(4.201–103.907)

*, *p*-value < 0.05.

**Table 3:** Dynamic variations in the studied microRNAs at one week after fecal microbiota transfer.

Variable	Pre-Transplant	Post-Transplant	*p*-Value
**miR-21**	32.61 (10.90–34.17)	12.83 (2.99–87.75)	0.021 *
**miR-122**	0.40 (0.01–0.50)	1.00 (0.62–1.00)	0.058
**miR-125**	0.49 (0.18–10.57)	1.00 (0.62–1.12)	0.062
**miR-146**	0.69 (0.02–0.92)	1.00 (1.00–1.21)	0.002 *
**miR-155**	0.40 (0.01–0.50)	1.00 (0.62–1.00)	0.068

*, *p*-value < 0.05.

**Table 4:** Multivariable linear regression with internal validation by bootstrapping between the relative expression of the studied micro-RNAs at one week post–fecal microbiota transfer and the variation in fibrosis markers.

Variable	B-Coefficient	*p*-Value	BCa 95%
**FIB-4**			
**miR-21**	−0.085	0.022 *	[−0.149, −0.020]
**miR-122**	−0.007	0.013 *	[−0.012, −0.002]
**miR-125**	0.012	0.686	[−0.053, 0.077]
**miR-146**	−0.004	0.001 *	[−0.006, −0.002]
**miR-155**	−0.007	0.013 *	[−0.014, −0.003]
**APRI**			
**miR-21**	−0.024	0.025 *	[−0.045, −0.04]
**miR-122**	−0.002	0.028 *	[−0.044, −0.001]
**miR-125**	0.012	0.171	[−0.006, 0.030]
**miR-146**	0.001	0.014 *	[−0.002, −0.001]
**miR-155**	−0.002	0.018 *	[−0.004, −0.001]
**E (kPa)**			
**miR-21**	−0.239	0.016 *	[−0.422, −0.056]
**miR-122**	−0.019	0.027 *	[−0.034, −0.004]
**miR-125**	0.047	0.578	[−0.134, 0.229]
**miR-146**	−0.011	0.004 *	[−0.017, −0.004]
**miR-155**	−0.019	0.034 *	[−0.034, −0.004]
**Cap (dB/m)**			
**miR-21**	−0.071	0.547	[−0.323, 0.181]
**miR-122**	−0.006	0.560	[−0.026, 0.015]
**miR-125**	−0.077	0.369	[−0.260, 0.102]
**miR-146**	0.007	0.126	[−0.016, 0.002]
**miR-155**	−0.007	0.595	[−0.026, 0.017]

*, *p*-value < 0.05.

**Table 5:** Pearson correlations between biological markers and the studied micro-RNAs at baseline.

Variable	miR-21	miR-122	miR-125	miR-146	miR-155
**CA 19-9**	−0.709 *	0.462	0.333	−0.085	0.316
**CEA**	−0.587 *	0.690 *	0.231	0.210	0.521
**AFP**	0.647 *	−0.276	−0.387	−0.427	−0.230
**Monocytes, count**	−0.371	0.606 *	0.598 *	0.337	0.458
**ESR**	−0.042	0.498	0.273	−0.024	0.681 *
**T4 free**	−0.296	0.362	0.331	0.670 *	0.206

ESR (Erythrocyte Sedimentation Rate); Values represent r_s_ (Spearman’s R) and significant values are flagged with (*, *p*-value < 0.05).

**Table 6:** Pearson correlations between biological markers and the fold change in the studied micro-RNAs at one week post–FMT.

Variable	miR-21	miR-122	miR-125	miR-146	miR-155
**Uric acid**	0.609	0.029	0.609	0.377	0.029
**Amylase**	0.257	0.600 *	−0.143	0.314	0.600 *
**Total bilirubin**	−0.657 *	0.143	−0.257	−0.429	0.143
**Cholesterol**	−0.200	−0.314	−0.029	0.600 *	−0.314
**Creatinine**	−0.600 *	−0.257	−0.543	−0.600 *	−0.257
**Iron-Serum iron**	−0.829 *	−0.257	−0.657 *	0.200	−0.257
**GGT**	−0.029	−0.829 *	−0.314	−0.657 *	−0.829 *
**HDL-Cholesterol**	−0.257	−0.143	−0.086	0.657 *	−0.143
**IgG**	0.086	0.429	0.371	−0.714 *	0.429
**IgM**	−0.029	0.086	−0.200	−0.714 *	0.086
**LDL-Cholesterol**	0.029	−0.086	0.200	0.714 *	−0.086
**Magnesium**	−0.714 *	−0.486	−0.771 *	0.257	−0.486
**CRP**	−0.029	−0.486	−0.086	−0.771 *	−0.486
**Total proteins**	0.441	0.500	0.794 *	−0.177	0.500
**ALT**	−0.943 *	−0.029	−0.543	0.029	−0.029
**INR**	−0.086	0.029	0.086	−0.714 *	0.029
**Leukocytes**	−0.657 *	0.143	−0.257	−0.429	0.143
**Hematocrit**	−0.200	−0.657 *	−0.143	0.200	−0.657 *
**MCV**	0.657 *	−0.371	−0.086	−0.029	−0.371
**Platelets**	0.200	0.429	0.600 *	−0.429	0.429
**MPV**	−0.086	−0.200	−0.600 *	0.029	−0.200
**PDW**	0.232	0.638 *	−0.116	0.464	0.638 *
**Macroplatelets**	0.232	0.406	−0.232	0.725 *	0.406
**Neutrophils**	−0.657 *	0.143	−0.257	−0.429	0.143
**Lymphocytes**	0.371	0.714 *	0.771 *	0.486	0.714 *
**NLR**	−0.371	−0.371	−0.429	−0.771 *	−0.371
**ESR**	0.543	0.429	0.829 *	−0.429	0.429
**Folic acid**	0.771 *	0.200	0.143	0.029	0.200
**AFP**	−0.800 *	0.300	0.100	−0.600	0.300
**Ferritin**	0.314	−0.371	−0.086	−0.714 *	−0.371
**Free T4**	−0.771 *	−0.086	−0.371	0.029	−0.086
**TSH**	−0.600 *	−0.029	−0.200	−0.486	−0.029
**Vitamin B12**	−0.029	0.086	−0.200	−0.714 *	0.086
**BMI**	−0.714 *	0.086	−0.086	0.086	0.086

ALT (Alanine aminotransferase), CRP (C-reactive protein), MCV (Mean Corpuscular Volume), MPV (Mean Platelet Volume), PDW (Platelet Distribution Width), BMI (Body Mass Index); Values represent r_s_ (Spearman’s R) and significant values are flagged with (*, *p*-value <0.05).

## Data Availability

The data presented in this study are available from the corresponding author upon reasonably request due to privacy, legal and ethical reasons.
